# Urinary Podocyte Loss Is Increased in Patients with Fabry Disease and Correlates with Clinical Severity of Fabry Nephropathy

**DOI:** 10.1371/journal.pone.0168346

**Published:** 2016-12-16

**Authors:** Brent Fall, C. Ronald Scott, Michael Mauer, Stuart Shankland, Jeffrey Pippin, Jonathan A. Jefferson, Eric Wallace, David Warnock, Behzad Najafian

**Affiliations:** 1 Department of Pathology, University of Washington, Seattle, United States America; 2 Department of Pediatrics, University of Washington, Seattle, United States America; 3 Departments of Pediatrics, University of Minnesota, Minneapolis, United States America; 4 Departments of Medicine, University of Minnesota, Minneapolis, United States America; 5 Department of Medicine, University of Washington, Seattle, United States America; 6 Department of Medicine, University of Alabama, Birmingham, United States America; Tokushima University Graduate School, JAPAN

## Abstract

Chronic kidney disease is a major complication of Fabry disease. Podocytes accumulate globotriaosylceramide inclusions more than other kidney cell types in Fabry patients. Podocyte injury occurs early in age, and is progressive. Since injured podocytes detach into the urine (podocyturia), we hypothesized that podocyturia would increase in Fabry patients and correlate with clinical severity of Fabry nephropathy. Urine specimens from 39 Fabry patients and 24 healthy subjects were evaluated for podocyturia. Most of the Fabry patients and many healthy subjects had podocyturia. The number of podocytes per gram of urine creatinine (UPodo/g Cr) was 3.6 fold greater in Fabry patients (3,741 ± 2796; p = 0.001) than healthy subjects (1,040 ± 972). Fabry patients with normoalbuminuria and normoproteinuria had over 2-fold greater UPodo/g Cr than healthy subjects (p = 0.048). UPodo/gCr was inversely related to eGFR in male patients (r = -0.69, p = 0.003). UPodo/gCr was directly related to urine protein creatinine ratio (r = 0.33; p = 0.04) in all Fabry patients. These studies confirm increased podocyturia in Fabry disease, even when proteinuria and albuminuria are absent. Podocyturia correlates with clinical severity of Fabry nephropathy, and potentially may be of prognostic value.

## Introduction

Fabry disease is caused by deficiency of the lysosomal enzyme alpha-galactosidase A, coded by GLA gene on Xq21.3-q22. Progressive renal failure is a major complication of Fabry disease. Enzyme replacement therapy (ERT) if initiated late in the course of the disease cannot prevent progressive decrease in the glomerular filtration rate (GFR).[1] Heterogeneity of Fabry disease phenotypes has hampered the establishment of a consensus guideline for when to initiate ERT. While some Fabry patients develop complications in childhood, others may only have minimal symptoms until adulthood. Biomarkers to detect early Fabry nephropathy, when lesions are more amenable to therapy, are therefore needed to individualize decisions for treatment initiation and assessment of treatment efficacy. Currently, proteinuria and microalbuminuria are commonly used for this purpose,[2] but, these are not sensitive to detect early Fabry nephropathy lesions[3, 4] and are not precise predictors of renal disease in female patients[5]. Podocytes have a limited capacity to regenerate[6] and podocyte injury and subsequent loss leads to segmental and global glomerulosclerosis[7], these indicative of irreversible nephron injury. In fact, segmental and global glomerulosclerosis are common findings in the later stages of Fabry nephropathy. Biopsy studies suggest that podocyte injury begins in early childhood and is progressive with increasing age.[3, 4] Thus, markers of podocyte injury are required to detect early Fabry nephropathy. While podocytes are more resistant to ERT-induced clearance from globotriasylceramide (GL3),[8, 9] the main substrate of a-galactosidase-A, a recent study suggested that early ERT initiation at 1mg/kg every 2 weeks may substantially reduce podocyte GL3 content in young patients,[10] this suggesting the importance of early treatment. Injured podocytes fall into the urine (podocyturia). Quantification of urinary podocytes (UPodo) thus provides robust evidence of podocyte injury. In fact, this parameter has been shown to have diagnostic and prognostic values in other glomerular diseases, such as preeclampsia, IgA nephropathy, and others.[11–14]. It is therefore likely that podocyturia in Fabry disease antedates and may well lead to proteinuria[15], glomerulosclerosis and reduced GFR. If true, podocyturia may be useful to predict the risk of Fabry nephropathy and guide treatment strategies. In this study, we developed and optimized cytospin techniques to quantify podocyturia in Fabry patients and compared the results to healthy volunteers.

## Results

### Demographical and Clinical Characteristics

Forty-nine patients with Fabry disease were enrolled (Table 1). One patient with a transplanted kidney was excluded and 9 other patients were excluded because of inadequate urine specimens. Therefore, the data presented here are from 39 Fabry patients (19 males and 20 females), age 44 [3–79], median [range]. The female patients in this study were, on average, 27 years older than the males (p = 0.0003) and their age of diagnosis of Fabry disease was, on average, 24 years greater than that of the males (p = 0.005). Twenty-five patients (64%) were normoalbuminuric [urine albumin/creatinine ratio, (UACR) < 30 mg/g], seventeen (44%) were normoproteinuric [urine protein/creatinine ratio, (UPCR) < 110 mg/g in males, and < 160 mg/g in females], and fifteen (39%) were both normoalbuminuric and normoproteinuric. Thirty-nine (80%) of patients were receiving a renin angiotensin system blocking medication at the time of study. Female patients had greater UACR (p = 0.01) and lower estimated GFR (eGFR) (p = 0.0003) than males. UPCR, systolic and diastolic blood pressures were not different between male and female patients. Aside from one patient whose ERT status could not be confirmed, 25 (64%) patients were receiving ERT (Table 2). Patients receiving ERT and those who were ERT-naïve were not different in sex distribution, age, UACR, UPCR or eGFR.

**Table 1:** Demographics and clinical characteristics of Fabry patients.

Case	Sex	Age	GLA mutation	age at Diagnosis	ERT	UACR	UPCR	eGFR
1	F	3	†	†	n	15	353	†
2*	F	30	R112C	†	y	15	83	120
3	F	36	c.966delC	33	y	608	844	96
4	F	38	q327x	36	y	437	561	106
5	F	40	†	13	y	7	93	†
6	F	42	†	18	†	43	188	†
7	F	43	†	20	y	14	96	95
8	F	44	†	18	n	16	375	111
9*	F	54	A97V	36	y	23	95	60
10	F	54	N224S	37	y	154	243	77
11*	f	54	†	†	y	†	†	83
12	F	56	†	15	n	13	182	98
13	F	57	R112C	14	y	30	93	71
14	F	58	C. 1041_1042 ins6/wt	56	y	2	57	70
15	F	64	†	40	n	20	86	93
16	F	64	m427	39	y	109	224	34
17	F	64	IVS6+2T>C	56	y	11	148	69
18	F	65	†	30	y	32	158	†
19	F	67	G261D	54	n	169	250	48
20	F	67	c.59_84del	34	n	243	341	†
21	F	67	E251X	†	y	535	1041	27
22	F	74	L300p	54	y	13	94	33
23	F	79	y151x	64	n	199	500	66
24*	M	7	†	6	y	20	600	211
25	M	13	18delA	5	n	12	82	162
26	M	13	†	3	y	8	129	†
27	M	14	c.59_84del	13	n	4	64	141
28	M	14	e.966delc	11	n	21	87	144
29	M	14	†	†	y	13	207	124
30	M	17	c.59_84del	15	n	4	32	133
31	M	19	97V	2	y	173	275	109
32*	M	20	†	†	†	†	†	†
33	M	22	c.11176>c(p.6373R)	17	y	2	40	134
34	M	29	C. 1041_1042ins6	28	y	8	240	112
35	M	31	R112C	9	y	16	94	117
36	M	33	n272k	10	y	9	94	133
37*	M	34	R112C	4	y	134	224	76
38	M	36	q327x	34	y	3	1803	60
39	M	42	†	10	y	5	176	†
40	M	44	†	†	n	10	176	124
41*	M	46	†	44	n	22	120	83
42*	M	49	†	†	†	1865	2262	†
43	M	50	7365 A>G	14	y	1	41	54
44	M	50	†	7	y	70	169	†
45	M	51	q250p	†	n	846	1088	106
46	M	52	†	51	y	4	92	101
47	M	54	†	†	y	7	113	105
48*	M	56	†	†	†	128	202	†
49*	M	58	†	†	†	†	†	†

*Excluded because of sample inadequacy (n = 10) or having a transplanted kidney (n = 1)

† data unavailable. Units: Age and age at diagnosis = year; UACR and UPCR = mg/g; eGFR = ml/min/1.73 m^2^.

Abbreviations: ERT = enzyme replacement therapy; UACR = urine albumin creatinine ratio; UPCR = urine protein creatinine ratio; eGFR = estimated glomerular filtration rate calculated by the CKD-EPI formula.[16]

**Table 2:** Comparison of clinical characteristics in male and female patients.

	Male (n = 19)	Female (n = 20)	P-value
Age (years)	31 [13–54]	58 [3–79]	0.0003
Age at diagnosis (years)	11 [2–51]	35 [13–64]	0.005
ERT-naïve (%)	68	60	NS
Systolic blood pressure (mmHg)*	134 ± 19	123 ± 12	NS
Diastolic blood pressure (mmHg)*	75 ± 8	74 ± 9	NS
UACR (mg/g)	8 [1–847]	31 [2–608]	0.01
UPCR (mg/g)	113 [32–1803]	206 [57–1041]	NS
eGFR (ml/min/1.73 m^2^)	116 ± 28	73 ± 28	0.0003

*Data available from 11 males and 12 females.

Abbreviations: ERT = enzyme replacement therapy; UACR = urine albumin creatinine ratio; UPCR = urine protein creatinine ratio; eGFR = estimated glomerular filtration rate

UACR in Fabry patients directly correlated with UPCR (r = 0.68; p = 0.0001). UACR showed a direct relationship with age (r = 0.33; p = 0.04), but there was no relationship between UPCR and age. When the analyses were done separately by sex, UACR and UPCR were correlated in both males (r = 0.51; p = 0.03) and females (r = 0.82; p = 0.0001). eGFR was inversely related to age in both male (r = -0.79, p = 0.0002) and female (r = -0.83, p = 0.0002) patients. UPCR showed an trend towards an inverse association with eGFR in male patients (r = -0.48, p = 0.06), but not in females.

Urine samples from 24 healthy volunteers, age 39 [6–63], M/F = 9/15 were used as normal controls. Male and female healthy volunteers were not different from each other in age, UACR or UPCR. UACR in healthy volunteers directly correlated with UPCR (r = 0.69; p<0.05), but there was no relationship between age and UACR or UPCR. Fabry patients were not statistically different from healthy controls for age, but had greater UACR (14 [1–847] in Fabry *vs*. 4 [0–125] in controls; p = 0.0006) and UPCR (169 [32–1802] *vs*. 65 [0–181] in controls; p = 0.0005).

### Urine Podocyte Loss is Increased in Patients with Fabry Disease

**Fig 1:**
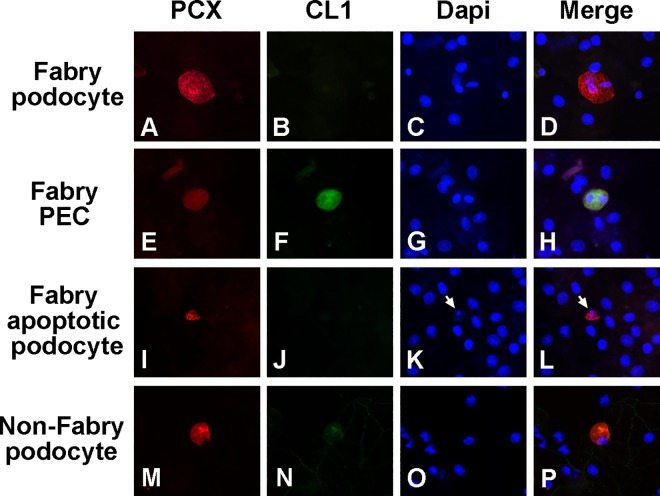
Urine cells on cytospin slides stained for podocalyxin (PCX, red), claudin-1 (CL1, green), and Dapi (blue). **(A-D)** A Faby podocyte which is PCX+/CL1- and shows characteristic vacuolated cytoplasm. **(E-H)** A urine cells which is PCX+/CL1+ consistent with a parietal epithelial cell (PEC) phenotype. **(I-L)** An apoptotic podocyte (PCX+/CL1-) in the urine from a Fabry patient with shrunken cytoplasm and small nucleus (arrow) compared to its adjacent cells. **(M-P)** A podocyte (PCX+/CL1-) in the urine from a healthy subject does not show vacuolated cytoplasm.

UPodo were found in the majority of Fabry patients (95%) and in healthy volunteers (92%). While UPodo were defined based on positive staining for podocalyxin (PCX) and negative staining for claudin-1 (CL1), the morphology of Upodo was not uniform (Fig 1A–1P); 35 ± 17% of UPodo in patients with Fabry disease and 30 ± 14% of these in healthy subjects (p = 0.21) had small shrunken/fragmented nuclei with apoptotic appearance (Fig 1I–1L), while the rest had normal appearing nuclei. Some of the non-apoptotic UPodo in Fabry patients were prominently larger in size than others and larger than UPodo in healthy volunteers. These larger UPodo had prominent cytoplasmic vacuolization, closely resembling the appearance of Fabry podocytes in-vivo (Fig 1A–1D).

**Fig 2:**
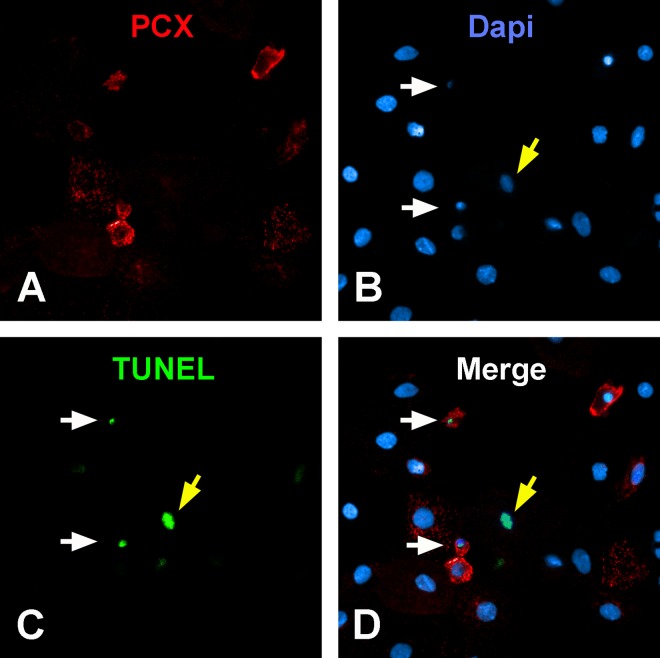
Urine cells on cytospin stained for podocalyxin (PCX, red), Dapi (blue), and TUNEL (green). Two podocytes (PCX+) with apoptotic nuclei (white arrows in B-D) are also positive for TUNEL (white arrows in C-D), confirming apoptosis. The yellow arrow (B-D) shows a non-podocyte cell (PCX-) that is TUNEL+ (C-D).

TUNEL staining performed in 5 urine samples from patients with Fabry disease confirmed positive nuclear staining, indicative of apoptosis, in 97% of the shrunken appearing nuclei. However, some of the urine cells with normal appearing nuclei also showed positive TUNEL staining (Fig 2). There were strong correlations between the number of apoptotic and non-apoptotic UPodo/gCr in the same urine in both Fabry patients (r = 0.71, p = 0.000001) and healthy subjects (r = 0.82, p = 000001).

**Fig 3:**
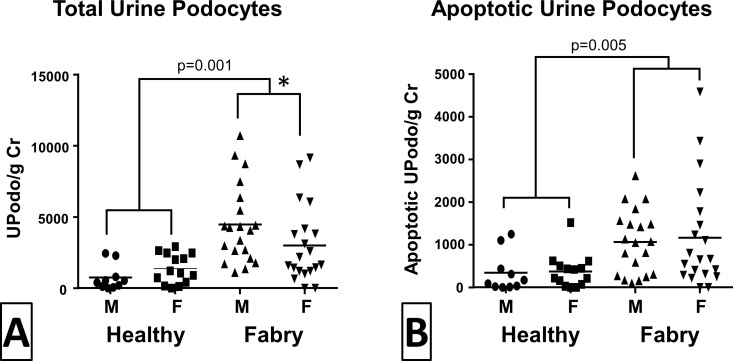
**(A)** Comparison of podocyturia in healthy subjects and Fabry patients. Fabry patients had significantly more urine podocytes (p = 0.001) per g creatinine (UPodo/g Cr). * There was a weak trend (p<0.1) for greater UPodo/g Cr in male Fabry patients compared to females. UPodo/g Cr was not different between male and female healthy subjects. **(B)** Comparison of urine apoptotic podocyturia in healthy subjects and Fabry patients. Fabry patients had significantly more apoptotic UPodo/g Cr than healthy subjects (p = 0.005). Apoptotic UPodo/g Cr were not different between males and females either in Fabry patients or in healthy subjects.

UPodo/gCr averaged 3.6 fold greater in Fabry patients (3,741 ± 2796) vs. healthy subjects (1,040 ± 972), p = 0.001 (Fig 3A). Similarly, Fabry patients had more apoptotic podocytes (1123 ± 1026 /gCr) than healthy subjects (363 ± 416 /gCr) (p = 0.005; Fig 3B). Importantly, normoalbuminuric and normoproteinuric Fabry patients had over twice as many UPodo/gCr than healthy subjects (2691 ± 2537 and 1046 ± 959, respectively; p = 0.048), while there was no age difference between these groups (data not shown). There was a weak trend for an association between UPodo/gCr and male sex (p<0.10) in Fabry patients. However, when the comparison was limited to podocytes with non-apoptotic appearing nuclei, UPodo/gCr in males (3,406 ± 2,197) was nearly twice that in females (1,845 ± 1,582, p = 0.01).

### Urine Podocyte Loss and Clinical Variables

In male but not in female Fabry patients, there were trends towards associations between age and total UPodo/gCr (r = 0.43, p = 0.06) and apoptotic UPodo/gCr (r = 0.43, p = 0.06). In healthy subjects, there was a trend towards an association between age and Upodo/gCr (r = 0.38, p = 0.07), which reached statistical significance when the analysis was limited to apoptotic UPodo/gCr (r = 0.43, p = 0.04).

**Fig 4:**
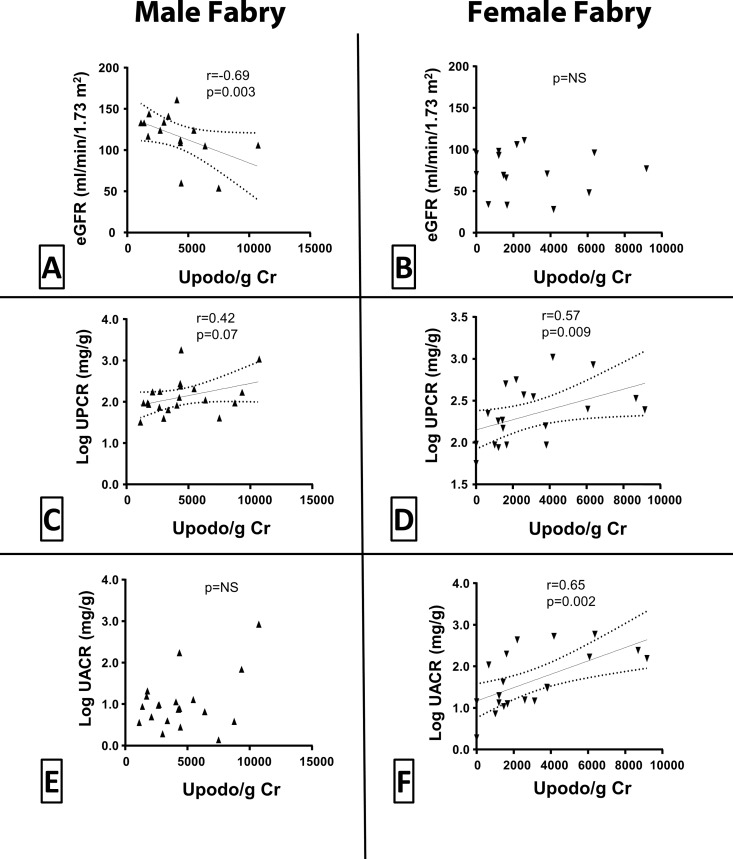
Relationships between podocyturia and renal function in patients with Fabry disease by gender. **(A and B)** Urine podocytes per g creatinine (UPodo/g Cr) vs. estimated GFR (eGFR) in males (A) and females (B). **(C and D)** UPodo/g Cr vs. urine protein creatinine ratio (UPCR) in males (C) and females (D). **(E and F)** UPodo/g Cr vs. urine albumin creatinine ratio (UACR) in males (E) and females (F). ns = statistically not significant.

UPodo/gCr was inversely related to eGFR in male patients (r = -0.69, p = 0.003), but not in females (Fig 4A and 4B). This relationship in males was present for both apoptotic (r = -0.57, p = 0.02) and non-apoptotic podocytes (r = -0.65, p = 0.006). UPodo/gCr was directly related to UPCR (r = 0.33; p = 0.04) in all Fabry patients (Fig 4C and 4D). However, this relationship was much stronger in females (r = 0.57, p = 0.009) than in males (r = 0.42, p = 0.07). Similarly, UPodo/gCr correlated with UACR only in female patients (r = 0.65, p = 0.002; Fig 4E and 4F). No relationship was found between UPodo/gCr and UACR or UPCR in healthy subjects.

There was no statistical difference between UPodo/gCr in Fabry patients who were receiving ERT (3,892 ± 2,798) and those who were ERT-naïve (3,712 ± 3,026). These results did not change when analyses were performed separately for male and female patients.

## Discussion

The most important findings in our study are that urinary podocyte loss is increased in Fabry patients and this correlates with clinical severity of Fabry nephropathy. Podocytes cannot efficiently replicate to compensate for their loss. Thus, podocyte injury can lead to progressive reduction in podocyte number in glomeruli and subsequent development of segmental and global glomerulosclerosis.[17] GL-3 inclusion accumulation in podocytes, similar to cardiomyocytes and corneal cells, starts in-utero [18] and podocytes accumulate more GL-3 than any other kidney cell in Fabry disease.[3] We previously showed that in young Fabry patients, podocyte GL-3 accumulation is progressive with age[3] and associated with foot process widening, consistent with podocyte injury, and both of these morphometrically measured electron microscopy parameters correlate with UACR. While quantitative measurements in biopsies such as reduced number of podocytes per glomerulus, foot process widening or podocyte detachment,[19, 20] are robust structural evidences of podocyte injury, podocyturia is a non-invasive indicator of podocyte loss Podocyturia in Fabry patients has been reported by others;[15, 21–23].Selvarajah *et al* reported PCX+ cells (*i*.*e*. podocytes) in the urine of half of the 35 Fabry patients they studied with no difference between those with or without Fabry nephropathy.[21] Trimarchi *et al*. reported podocyturia in a young Fabry patient without proteinuria, suggesting that podocyturia may antedate proteinuria.[23] Consistent with this observation, the results of the current study shows that Fabry patients with normal urinary albumin and protein excretion have over twofold urinary podocyte loss compared to healthy control subjects of comparable age. These findings are consistent with biopsy studies demonstrating that podocyte injury antedates proteinuria in Fabry patients. [3, 4] Proteinuria is not a sufficiently sensitive biomarker to detect early and progressive kidney injury in Fabry nephropathy. Moreover, once proteinuria is established, ERT may not prevent renal functional decline.[9, 24] Given the heterogeneity of phenotype severity in Fabry patients,[25] an early non-invasive indicator of the kidney injury to identify patients at greater risk of progression to CKD, who might benefit from higher doses of ERT,[26] is needed. This need is even greater in female patients, where heterogeneity in phenotype severity is even greater, perhaps in part, related to mosaicism of podocyte involvement.[27] In another study, Trimarchi *et al*. found increased podocyturia in Fabry patients, but with no correlation between urine podocyte density and either proteinuria or eGFR.[15] In contrast, Pereira et al. found correlation between podocyturia and UACR in Fabry patients. [22] The findings of the current study are consistent with Pereira et al. with direct correlations between podocyturia and proteinuria in both male and female patients. Importantly, we found an inverse correlation between podocyturia and eGFR in male patients, suggesting that deteriorating renal function in Fabry nephropathy is associated with increased podocyturia. We suspect that the discrepancies observed among the above studies may, at least in part, be related to different methodologies used in these studies, as well as differences in studied patient populations. Future studies should also be directed at standardization of podocyturia assessment with the ultimate goal of developing a test meeting the regulatory requirements for clinical application.

It should be noted that although podocyturia may potentially be of use to identify Fabry patients with more severe nephropathy, it is not, per se, specific for Fabry disease. On the other hand, Selvarajah et al found that CD77 (also known as GL-3)+ vacuolated cells are frequently found in urine from Fabry patients and suggested that this might be a sensitive and specific tool to identify the patients. While, it is likely that these cells observed by Selvaragah et al were podocytes, they did not perform dual labelling of podocyte marker (podocalyxin) and CD77. Moreover, podocalyxin+ cells (i.e. podocytes) were less frequent (~50%) than with CD77+ cells in urine from Fabry patients. Consistent with this observation, we found that many, but not all, of Fabry podocytes had a vacuolated appearance. The focus of our study was quantification of podocytes in the urine. For this, we performed dual labelling with one inclusion marker (podocalyxin) and one exclusion marker (claudin-1 which is a tight junction molecule specific for parietal epithelial cells in glomeruli[28]). We did not add CD77 staining to our panel as it would have made our immunostaining more challenging. Nevertheless, although identification of CD77+ podocytes may potentially be a useful screening tool to identify Fabry patients, we believe that currently available standard screening tools (leukocyte a-galactosidase A activity and sequencing) are in general more appropriate for this purpose.

Podocytes are far more resistant than most other cell types to clear from GL3 inclusions following ERT.[8, 9]; although recent reports are indicative that a few years of ERT can clear podocytes from GL-3 inclusions in younger patients[10, 29, 30] Since glomerular podocyte loss is thought to be irreversible, amelioration or halting of podocyturia may be a reasonable indicator if a given treatment benefits podocyte, and ultimately renal survival. Trimarchi *et al*. reported greater podocyturia in ERT-naive adult Fabry patients compared to those on ERT; [15] however, the ERT-naïve patients in this study had lower proteinuria and higher GFR than those receiving ERT, indicative of less severe nephropathy in the ERT-naïve patients. In contrast, in the current study, where ERT-naïve and ERT-receiving patients were not different in age, proteinuria, albuminuria or eGFR and thus had more comparable nephropathy, there was no statistical difference in podocyturia between the groups. Consistent with our findings, Selvarajah et al. did not find difference in podocyturai between those who received ERT and those who did not;[21] however, proper assessment of ERT effect on podocyturia would require longitudinal studies to be done in the future. Also, and equally important, would be longitudinal studies of podocyturia and treatment outcomes to determine if this parameter may serve as a surrogate biomarker to predict hard outcomes. This approach is supported by studies showing that podocyturia correlated with biopsy severity and disease progression in various forms of glomerulonephritis, including IgA nephropathy, lupus nephritis, diabetic nephropathy, etc.[12, 31–34].

Apoptosis is a known mechanism of podocyte injury and loss.[35] Vogelmann *et al*. reported apoptotic podocytes in urine samples from patients with active focal and segmental glomerulosclerosis or lupus nephritis.[36] *In-vitro* studies suggested that dysregulated autophagy leads to apoptosis and podocyte loss in Fabry disease.[37] Supportive of this notion, the current study showed far greater numbers of apoptotic podocytes in urine samples from Fabry patients compared to controls, although fraction of podocytes that appeared apoptotic was not different between the groups. Recently, Hodgin *et al*. showed reduced glomerular podocyte density with aging.[20] Aging is associated with increased cellular apoptosis; however, apoptotic podocytes are uncommon in glomeruli,[38] likely due to their rapid detachment. Our study which showed a significant correlation between urinary apoptotic podocytes and age in healthy subjects, suggests that apoptosis may play a role in podocyte loss associated with aging. This introduces the idea that aging represents a second hit to the podocytes in Fabry disease or in other chronic kidney diseases associated with podocytopathy.

In summary, this cross-sectional study shows that Fabry disease is associated with increased podocyte loss. The direct associations found between podocyturia and proteinuria and the inverse association found between podocyturia and eGFR in male patients indicate that there are important correlations between podocyturia and severity of Fabry nephropathy. The methodologies we introduced in this work, especially the protocol for urine preservation that allows shipping of the specimens, can be instrumental in studying podocyturia in multi-center studies, not only in Fabry disease, but also in other kidney diseases.

The current study faces certain limitations. Females enrolled in this study were older and overall had more severe clinical Fabry nephropathy, these differences limiting the value of the comparisons by sex in the present study. This prominent age difference might have been in part due to the fact that many older male patients would have already progressed to advanced CKD with a need to dialysis or transplantation, conditions which would have excluded those from enrollment into the study. The study was cross-sectional with no paired samples obtained prior and after ERT, this hampering our ability to sufficiently assess the effect of ERT on podocyturia. For the same reason, addressing predictive value of podocyturia for Fabry nephropathy progression was not possible. Correlative biopsy studies will be important to compare podocyturia with reduced glomerular podocyte number in-vivo. Similar to any other biomarker, extensive studies to fulfill FDA approval criteria are needed to be done, such as those showing reproducibility, sensitivity, detection limits, etc. Sufficient knowledge about day to day variation of podocyturia in health and in Fabry disease is not available and will be necessary before taking this assay to clinical grounds.

## Methods

### Subjects and controls

The study procedures followed were approved by the Human Subject Division of the University of Washington, the Institutional Review Board of the University of Minnesota, and Office of the IRB—University of Alabama at Birmingham and in accordance with the ethical standards of the Responsible Committee on Human Experimentation (institutional and national) and with principles of the Declaration of Helsinki 1975, as revised in 2000 [39]. All patients gave IRB-approved written informed consent prior to enrollment into these studies. Fabry patients were recruited either during the clinical visits or in conjunction with Fabry disease support group meetings. Inclusion criteria included confirmed diagnosis of Fabry disease by GLA mutation analysis or abnormally low leukocyte α-Gal-A activity. Relevant clinical information was extracted from medical records. Healthy volunteers with no known kidney disease, hypertension, diabetes, or history of systemic disease or smoking were recruited through online volunteer websites at the University of Washington.

Urine albumin was measured by turbidometry, and urine total protein and creatinine were measured by spectrophotometry in the clinical laboratory of University of Washington Medical Center. Serum creatinine values were extracted from electronic medical charts and estimated GFR (eGFR) was calculated using the CKD-EPI[16].

### Specimen collection and processing

Random spot urine specimens were collected in sterile cups. One ml of urine was stored at -80c for later measurement of albumin, total protein and creatinine. The volume of the remainder was recorded. All specimens were centrifuged at 500 ×g for 6 minutes at 5°C in 50 ml conical tubes. The supernatant was slowly discarded. For urine samples that were collected in locations other than University of Washington (n = 8), the pellet was re-suspended in the minimal volume of urine remaining at the bottom of the 50 ml conical tube after discarding the supernatant (approximately 250 μl) and then mixed 1:1 with Streck Cell Preservative (Streck, Omaha, NE), transferred to a cryotube, and held at 4°C until it was shipped to University of Washington within a maximum of 3 days. This protocol preserves morphology and antigen expression (for podocalyxin and claudin-1) for a minimum 5 days (data not shown). Once these received, sample volumes were raised to 10 ml by adding a solution of RPMI-1640 (Invitrogen) with 3% fetal bovine serum (Sigma-Aldrich) and 1mM EDTA (Affymetrix). Similarly, for urine specimens that were collected at the University of Washington (n = 41) and processed fresh, the pellet was re-suspended in 10 ml of the RPMI cocktail followed by centrifugation at 500 × g for 6 min at 5°C. The rest of the processing was the same for samples that were shipped or processed freshly. The re-suspended pellet was diluted to a cell density not exceeding 2.5 x 10^5^ cells per ml (checked by hemocytometer), this the recommended specimen cell density by the manufacturer (Shandon, Thermo Scientific) to provide a monolayer of cells on cytospin slides. The diluted suspension was distributed in 200 μl volumes among the double spot cytofunnels (Shandon, Thermo Scientific) with charged double circle cytospin slide inserts. The number of cytospin slides used and the excess suspension volume were recorded. A Shandon Thermo Scientific Cytospin 4 was used at 1000 rpm for 12 minutes to prepare the cytospin slides. The slides were air dried modestly, then fixed in cold pure methanol and stored at -20°C until stained for immunofluorescence studies.

### Immunofluorescence Studies

Podocalyxin[40] (PCX) is a highly negatively charged molecule which is abundantly expressed on the surface of podocytes and hence is a sensitive marker of these cells commonly used in podocyturia studies[13, 41, 42]; however, since some of the parietal cells covering Bowman's capsule also express podocalyxin[42], we used claudin-1 (CL-1) which, in glomeruli, is specifically expressed in parietal epithelial cells, as an exclusion marker. The cytospin slides were stained with antibodies against PCX (Invitrogen, Camarillo, CA. 39–3800, Mouse monoclonal) at 1:50 dilution, and CL-1 (Invitrogen, Camarillo, CA. 71–7800 Rabbit polyclonal) at 1:50 dilution. Primary antibodies were labeled with fluorescent labeled secondary antibodies Alexa Fluor 594 goat anti-mouse IgG for anti-PCX antibody (Invitrogen Camarillo, CA., A11005) and Alexa Fluor 488 goat anti-rabbit IgG for anti-CL-1 antibody (Invitrogen Camarillo, CA A11008), both at 1:500 dilution. The stained cytospin slides were mounted with Prolong Gold anti-fade reagent with DAPI (Invitrogen, P36931), and kept at 4°C until scanning. Stained slides were scanned with 20x objective on an Aperio Scanscope FL slide scanner. Three separate images were captured for each spot utilizing filters corresponding to DAPI/Alexa350, FITC/ Alexa 488 and TXRed/Alexa 594 staining for later image fusion/analysis.

### Quantitation and Scoring:

Virtual Aperio scanned slides were examined at 20X magnification using the Aperio Imagescope Viewer (Leica Biosystems). All nucleated cells (DAPI+) were annotated for PCX (Alexa 594) staining, and all nucleated cells that were PCX+ were annotated for CL-1 (Alexa 488). PCX+/CL-1- cells were considered to be podocytes. Background staining was determined by using only secondary antibody stained slides that were prepared at each staining session and were scanned along with the same batch. Since some nuclei appeared small/apoptotic, the cells were also annotated for size of the nuclei (large *vs*. small).

Knowing the original urine volume, the number of cytospins utilized and the excess urine volume after distributing the suspension into the cytospin funnels, the urine volume represented in each cytospin was calculated, and the number of podocytes per ml urine and per urine Cr (UPodo/gCr) were calculated, thereof.

### Statistics

Variables were expressed as mean ± SD, except for age and UACR and UPCR which, not normally distributed, were expressed as median [range]. Group comparisons were done using Kolmogorov-Smirnov test. Correlations between variables were examined using Spearman’s rank order test. P<0.05 was considered statistically significant.
